# Topics of Public Interest

**Published:** 1915-11

**Authors:** 


### TOPICS OF PUBLIC INTEREST

**Three Cent Fares in Arkansas.** Federal Judge Jacob Fisher has granted continuance till April 17, 1917, on injunctions by the State R. R. Commissioners against three-cent fares.

---

**Effect of War on Immigration.** For the fiscal years ending June 30, immigration reached the million mark in 1905 and averaged about a million until 1914. For 1915, the immigration was 326,700, rather less than the average from 1850 to 1880, except for a marked reduction corresponding to the Civil War. It is not generally realized that, apart from ordinary travel, our country is subject to a very considerable emigration of aliens and others, mostly recently naturalized citizens. For the year 1912-13, the immigration was 1,197,892 and the emigration 611,924, net increase 815,303. For 1913-14 the respective numbers were 1,218,480; 633,805; 769,276. For 1914-15, 326,700; 384,174; 50,070. The last number represents the net gain for the reason that non-immigrant aliens become relatively important in the last year, whereas in previous years they are lost in the bulk of immigrant aliens. For July and August, 54,510 persons entered and 57,752 left the country, not counting ordinary travel.

